# DKCDC: A clustering algorithm focusing on genuine boundary search for regional division

**DOI:** 10.1371/journal.pone.0331555

**Published:** 2025-09-04

**Authors:** Qin Zheng, Keju Zhang, Qianqian Chen, Jianwei Wu, Jiaxiang Lin

**Affiliations:** 1 Key Laboratory of Smart Agriculture and Forestry, Fujian Agriculture and Forestry University, Fuzhou, Fujian, China; 2 Third Institute of Oceanography, Ministry of Natural Resources, Xiamen, Fujian, China; University of Calabar, NIGERIA

## Abstract

The majority of existing clustering algorithms, including those algorithms that focus on boundary detection, seldom account for the reasonableness and genuineness of boundaries, consequently, it is difficult to obtain well-defined boundary in clustering-based regional division. A novel boundary search Clustering algorithm integrating Direction Centrality with the Distance of K-nearest-neighbor (DKCDC) is proposed, which is capable of achieving well-defined regional boundaries, to resolve the challenges mentioned above. Firstly, the preliminary boundary of clusters are established on the basis of boundary points and initial cluster labels obtained by the Clustering algorithm using the local Direction Centrality (CDC). Secondly, all the boundary points are further processed and discriminated, to detect noise points concealed within the boundaries, which provides the essential basis for achieving more genuine and reliable cluster boundaries and regional identification. In this process, a fusion strategy is adopted, to subdivide the boundary points into true boundaries and false boundaries by combining voting method and distance metric. Thirdly, a regional division result with well-defined boundary is obtained by DKCDC. In the end, by distinguishing genuine from false boundaries using fusion strategy, DKCDC enhances regional boundary demarcation. Experiments on synthetic and UCI datasets show DKCDC improves silhouette coefficient by at least s4.88% over CDC, K-Means, DBSCAN, OPTICS and HDBSCAN, indicating its broad potential for applications in clustering-based regional division.

## Introduction

Clustering algorithm is one of the most important unsupervised learning methods [1]. The objective of clustering is to categorize unlabeled data objects into multiple groups, ensuring that data within the same group are closer to each other than those in different group [2]. Clustering algorithms are often combined with anomaly detection to make clustering results more accurate by detecting anomalies in the datasets. Until now, clustering algorithms have been widely used in different fields such as Market Segmentation [3], Image Segmentation [4,5], Document Analysis [6], Social Network Analysis [7] and Genetic Data Analysis [8].

Clustering-based regional division plays a critical role in practical applications. In 2021, Deng et al. [9] used clustering method to regionalize China’s office building climate. This study employed K-Means and agglomerative hierarchical clustering techniques to analyze the obtained normalized heat load data, aiming to formulate a zoning scheme and reveal the true energy demand. Scitovski [10] considered the possibility of applying density-based clustering algorithms to earthquake zoning. Through the use of density-based clustering in earthquake zoning, non-convex shapes can be identified, and the optimal number of clusters can be determined automatically without relying on indices, resulting in more realistic outcomes. Vuuren et al. [11] applied a clustering large applications algorithm, which is based on the partitioning around mediods algorithm, to cluster the temporal wind speed profiles associated with the South African renewable energy development zones. The resulting clusters greatly reduces the computational cost of high-level capacity allocation optimization studies. Kim et al. [12] performed a cluster analysis on wave observation and model data using the K-Means algorithm, to check the validity of the current forecast zone for the wave forecast. SHI et al. [13] presented a new zoning method using K-Means algorithm. The clustering result obtained by K-Means is used for the environmental risk zoning, and the zoning result is mapped using the geographic information system. As the result, the zoning is helpful in risk management and is convenient for decision makers to distribute limited resources to different sub-areas in the design of risk reducing intervention. Griffone et al. [14] presented the application results of K-Means, K-Medoids, hierarchical clustering and price differential clustering in forming bidding zones. The clustering algorithms have been applied to the study of a reduced model of the European transmission system, for different numbers of clusters. Genuine and reliable regional division has brought a certain degree of convenience to life and production, therefore, clustering algorithms based on boundary detection showed substantial theoretical significance and practical value in regional division.

With the advancement of research in clustering algorithms, numerous distinct algorithms have been proposed sequentially. Nonetheless, the majority of current clustering algorithms generally neglect the assessment of reasonableness and genuineness regarding cluster boundaries. Even algorithms such as the CDC [15] and the AUTOCLUST [16] series, which based on boundary point detection, primarily aim to disrupt connections between boundary points of different clusters through boundary identification to improve clustering accuracy. However, as these algorithms are not primarily aimed at regional division, it is inevitable that the genuineness of clustering boundaries will be overlooked, which results in the inability to detect potential noise within boundary points, thus, it is difficult to produce clustering results with well-defined boundary.

Therefore, this paper proposes a DKCDC algorithm for region recognition that enhances boundary accuracy by precisely identifying noise within boundary points. Based on boundary points obtained from KNN distribution, the DKCDC algorithm implements a fusion strategy that combines voting method and distances method to detects noise within boundary, including bridge points and points slightly deviated from cluster centers. Therefore, this method can effectively interrupt the connection between different clusters connected by bridges and avoid the interference caused by deviation points, making the boundary of region more clearer. The main innovations and contributions are summarized below:

- DKCDC separates boundary points into noise and normal points through a fusion strategy combining the number of votes and the K-nearest neighbor distance, thereby obtaining well-defined clustering boundary.
- The subjective influence caused by the artificial selection of parameter *r* value in the fusion strategy can be effectively avoided, through setting an adaptive parameter adjustment function.
- The proposed algorithm in this paper is tested on artificial datasets and UCI datasets, and its performance is compared with several representative clustering algorithms. The experiments show that DKCDC improves silhouette coefficient by at least 4.88% over CDC, K-Means, DBSCAN, OPTICS and HDBSCAN.

## Related works

To highlight the differences between the proposed algorithm and existing clustering algorithms, in this section, the advantages and disadvantages of different types of clustering algorithms are systematically reviewed and compared, besides, its application in regional division was discussed.

Partition-based clustering, which is a type of algorithms that divides a dataset into a specified number of clusters, with each cluster being defined by the mean of the points it contains. The advantages of this type of algorithm are simplicity and speed, but its disadvantage of being sensitive to noise [17] often leads to poor clustering results. Common algorithms include K-Means [18], K-Means++ [19] and K-Medoids [20] algorithms. As a typical partition-based clustering algorithm, K-Means divides the dataset into K clusters through continuous iteration, and each cluster is defined by the mean of all points in its cluster [21]. The characteristics of K-Means algorithm make it have better results for clustering spherical clusters, furthermore, K-Means has been widely used in scientific and industrial fields due to its simplicity, low time consumption, and low space complexity [22]. However, K-Means does not exclude any data point; instead, it assigns every point, including very remote points (deviant points), to the nearest centroid, even if such assignment is not appropriate. Therefore, under the K-Means algorithm, noise points will be regarded as normal points to classified into a cluster, and cannot be detected, thus affecting the clustering results. Qi [23] used the K-Means algorithm to carry out the regionalization of cultivated land intensive utilization in Hubei province. However, the limitation of K-Means in identifying outliers, results in challenges in achieving genuine clustering boundaries. Consequently, in the application of cultivated land intensive utilization zoning, the algorithm fails to accurately division outlier regions, leading to the cultivated land cannot be utilized rationally.

Density-based clustering, it is defined by areas in which the density of the data points is high, and clusters are separated from each other by areas of low density [24]. This type of algorithm can better identify clusters of any shape and can handle noise points and outliers [25]. However, it is sensitive to local density changes in the data, resulting in the inability to correctly identify clusters with weak connectivity. DBSCAN [26] algorithm is a typical density-based clustering. This algorithm contains two key parameters eps and minpts [27], the core points, boundary points and noise points are marked based on these two parameters, and then the clusters are divided by continuously constructing and expanding the core neighborhood. DBSCAN can effectively handle clusters of complex shapes and it is insensitive to noise, so it can identify outliers. However, when a bridge connection exists between two clusters, the bridging structure enhances the connectivity of the two clusters. If the points on the bridging structure satisfy the eps neighborhood conditions and accumulate a sufficient number of neighboring points (minpts), they will be mistakenly classified as core points or boundary points, resulting in the two distinct clusters may be erroneously merged. Since the points on the bridging structure cannot be detected as noise points, the connection between the two clusters cannot be interrupted. OPTICS [28] is a density-based clustering algorithm that addresses the limitations of DBSCAN in datasets with significant differential density. The core idea of the algorithm is to reflect the density relationship of data points in the local space by introducing two important concepts: core distance and reachable distance, and organize this information into a reachability graph to observe the potential clustering structure in the data. However, OPTICS mainly relies on the reachability plot to reflect the clustering structure, which makes it easy to mislabel boundary points with high reachability distances at the edge of sparse clusters as noise. HDBSCAN [29] is an extension of the DBSCAN and OPTICS algorithms, aiming to overcome the limitations of density-based clustering in handling with non-uniform density cluster structures. The core idea of the algorithm is to perform agglomerative hierarchical clustering by constructing a density-reachable graph and applying a minimum spanning tree, then automatically extract the most representative clustering structure through an “optimal flattening” process based on cluster stability. However, HDBSCAN uses density reachability and cluster stability as core criteria. It mainly focuses on whether a data point belongs to a cluster, but ignores the judgment of whether the data point is at the edge of the cluster, resulting in its inability to identify cluster boundaries. Karri et al. [30] applied a density based clustering algorithm (DBSCAN) to the earthquake point data of India and thereby divides the region into the various seismic zones depending on the analysis of the various clusters formed. However, since DBSCAN does not account for the genuineness of clustering boundaries, it is challenging to achieve reliable seismic zone division for areas located near the boundaries in seismic zoning applications.

Hierarchy-based clustering, it is a strategy of cluster analysis to create a hierarchical of clusters [31]. The advantage of this type of algorithm is that it can discover clusters of any shape and scale, but its computational complexity is high and is not conducive to clustering large datasets. Furthermore, hierarchy-based clustering lacks the ability to handle noise points. It assumes that all points belong to some cluster and construct a tree-like clustering structure by recursively merging or splitting data. As a result, every data point, including outliers and remote points, is forcibly assigned to a cluster, thus affecting the clustering results. There are two main types of hierarchical clustering, bottom-up agglomerative hierarchical clustering [32] and top-down divisive hierarchical clustering [33]. To gain a deeper understanding of the cultural heritage protection and urban planning of the Weibei Imperial Mausoleum Protection Zone, Feng [34] constructed a settlement spatial distribution model based on factor analysis combined with a hierarchical clustering algorithm, which provided valuable insights for urban planning and cultural heritage protection. However, due to the high time complexity of the hierarchical clustering algorithm, it may not be able to obtain satisfactory results on longer datasets.

Grid-based clustering, it is a method that divides the data space into a finite number of grid cells [35] and then performs clustering on these grid cells. This type of method demonstrates significant effectiveness in handling large-scale data, but it is more suitable for discovering clusters with regular shapes and is less effective for clusters with complex shapes. Although grid-based clustering can ignore or identify low-density grid cells as noise according to the density threshold, the size of the grid cells depends on the artificially set threshold, and unreasonable grid division will lead to misjudgment of noise points. Chen et al. [36] compared the improved K-Means clustering algorithm with the grid-based clustering algorithm, a batch-clustering algorithm is also developed to minimize the computational time and resources associated with simulating earthquake damage processes in large urban building complexes. The study showed that the improved K-Means algorithm is more suitable for simulating earthquake damage processes.

Therefore, to address the defect that most algorithms cannot identify genuine boundaries, this paper proposes a boundary search clustering algorithm called DKCDC, which has the purpose of regional division. A new fusion strategy is employed on the boundary points obtained from the KNN distribution. This strategy effectively identifies bridge points and points slightly deviated from cluster centers, which are contained within the boundary, as noise, leading to obtain well-defined clustering boundary.

## Methods

### Existing boundary-based cluster algorithm

Unlike traditional methods, boundary-based clustering algorithms disrupt the connections between boundary points of different clusters by detecting boundary outline, especially in datasets with irregular shapes and varying densities. It has become one of the core technologies in modern spatial data mining. This section introduces two typical boundary-based clustering algorithms.

AUTOCLUST is a boundary detection clustering algorithm using Delaunay graphs. The core idea is to discover clusters by locating their boundary using a Delaunay graph, and removing connections between boundary points of different clusters, relying on the fact that boundary points usually exhibit high standard deviations in incident edge lengths. However, the boundary identification of AUTOCLUST relies on the comparison of the local standard deviation of the edge length with the global average standard deviation, which makes it easy to misidentify isolated points as internal points and fail to break the bridging structure.

The CDC algorithm, which is centered on boundary detection, proposes a local directional centrality measure (DCM) based on the difference in neighborhood distribution characteristics between internal points and boundary points of the cluster to represent the variance of the angle formed by K-nearest-neighbors. Among them, internal points have lower DCM values, while boundary points have higher DCM values. Therefore, internal points and boundary points can be divided by the parameter *T*_*DCM*_. Subsequently, the outer contour constructed from the identified boundary points constrains the connections among internal points, effectively preventing cross-cluster connections. Therefore, it has better clustering results for datasets with heterogeneous density and weak connectivity.

However, the CDC algorithm only considers the differences in neighborhood distribution characteristics between internal points and boundary points, and divides the points in the neighborhood into boundary points and internal points, but ignores the existence of noise points in the neighborhood, resulting in the noise hidden in the boundary cannot accurately be detected, making it difficult to obtain a more reasonable clustering boundary. Particularly, when bridging structures or deviation points exist between clusters, since the deviation points and the points on the bridging structure only include neighboring points within a certain direction range, the bridging structure or deviation points will also be classified as boundary points and assigned to the nearest cluster. This irrational division of cluster boundary will result in the inability to interrupt the bridge connections between clusters, and it will also be impossible to avoid the interference caused by deviation points, thus affecting the clustering results.

Therefore, this paper proposes a new boundary search clustering algorithm using a fusion strategy. This novel algorithm corrects the inability of the CDC algorithm, as well as most existing methods, to correctly identify boundaries by accurately detecting noise within the boundary points, thereby obtaining more genuine and reliable boundaries and improving the performance of clustering.

### Core idea of DKCDC

**Fig 1:**
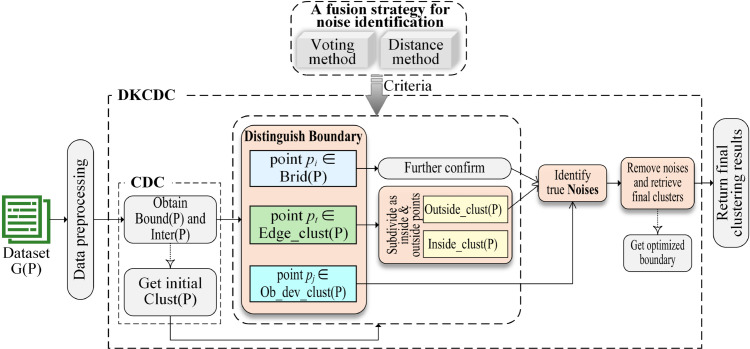
Technical route of DKCDC algorithm. *Bound*(*P*) and *Inter*(*P*) denote the set of boundary point and the set of internal point respectively. *Clust*(*P*) denotes the set of initial clustering label obtained by CDC. *Brid*(*P*) denotes the points on the bridge. Ob_dev_clust(P) denotes the points that are obviously deviated from the cluster. Edge_clust(P) denotes the points on the edge of the cluster. Outside_clust(P) denotes unobvious deviation points at the edge of clusters. Inside_clust(P) denotes interior points at the edge of the clusters.

Fig 1 briefly summarizes the technical route of the DKCDC algorithm. The initialization method of DKCDC is as follows: given the original dataset *G*(*P*), data preprocessing is first conducted, which involves imputing missing values using the mean and standardizing the data; Then, the traditional CDC algorithm is used to divide the set of boundary points *Bound*(*P*) and the set of internal points *Inter*(*P*) based on the KNN distribution of data points to obtain a rough boundary; Last, the internal points and boundary points are clustered to obtain the initial cluster labels *Clust*(*P*). Since the CDC algorithm can effectively prevent cross-cluster connections, internal points can be assigned to the correct cluster labels. However, the CDC algorithm cannot detect noise points, result in it is unable to handle the noise contained in the boundary points, making it difficult to obtain a more genuine and reliable boundary. Particularly for clusters connected by bridges, points on the bridges and points that deviate from the clusters, which would be more appropriately classified as noise, are often incorrectly identified as in-cluster points, resulting in unsatisfactory clustering results. Therefore, the next stage requires in-depth research on boundary points, and the more accurate clustering results at the boundary can be obtained, through accurately identifying noise points, thereby enhancing the overall clustering quality.

**Fig 2:**
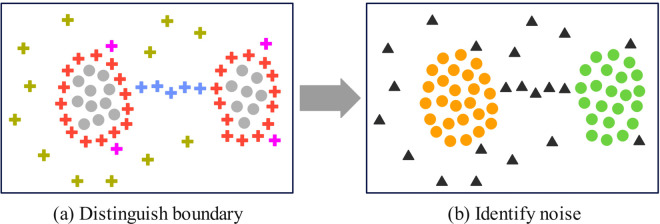
The geometric meaning of bridge points, deviation points and less deviated edge points.

To achieve genuine clustering boundaries, this study proposes a DKCDC algorithm, which accurately identifies noise concealed within boundaries. This algorithm adopts a fusion strategy that combines the voting method and the distance method, to conducts in-depth research on the boundary points obtained by KNN distribution partitioning. Based on the obtained *Bound*(*P*), the fusion strategy is adopted to distinguish the boundary points into points on the bridge, denoted as $p_{i}\in Brid(P)$, points that are obviously deviated from the cluster, denoted as $p_{i}\in Ob\_dev\_clust(P)$, and points that are less obviously deviated at the edge of clusters, denoted as $p_{i}\in Edge\_clust(P)$. The geometric meaning of these points is shown in Fig 2(a). The blue points in Fig 2(a) represent bridge points, which are located on the bridging structure connecting two clusters. Geometrically, these points form part of the bridging structure, and their local directional consistency may present a linear structure. As a result, the incorrect merging of clusters that should have been separated. The green points in Fig 2(a) denote deviation points, which are points that are obviously deviated from the cluster and exhibit obvious differences from the cluster density distribution. Such points may be irregular extensions of the boundaries, which can easily affect the regional shape of the cluster. The purple points in Fig 2(a) represent less deviated edge points, which are located at the edge of the cluster and slightly deviating from the cluster density distribution. These points remain within the effective boundary of the cluster, although their density is slightly lower than that of the core region. Therefore, the bridge points, deviation points and less deviated edge points are invalid boundary points, that is, false boundaries, and should be removed from the original cluster as noise points. The red points in Fig 2(a) are valid boundary points, that is, true boundaries, and should be returned to the corresponding clusters. The results are shown in Fig 2(b). The true boundary is returned to the corresponding cluster, while the false boundary are identified as noise points (black triangles). As a result, the two clusters connected by the bridge are effectively separated.

The points that are obviously deviated from the cluster are directly identified as noise, and points on the bridges are also identified as noise through further confirmed. While, the points that are less obviously deviated at the edge of clusters are subdivided as Inside_clust(P) and Outside_clust(P), and the points in Outside_clust(P) are identified as noise. A genuine boundary is obtained to correct the clustering errors caused by inaccurate boundaries, through identifying the noise points hidden in the boundary. In addition, to improve the reliability of the overall clustering results, small clusters with a number of sample points less than a certain threshold are also handled. Finally, the true boundaries are returned to the corresponding clusters, and the false boundaries which are identified as noise are removed from the original clusters, based on the obtained genuine boundaries and initial clustering labels.

**Fig 3:**
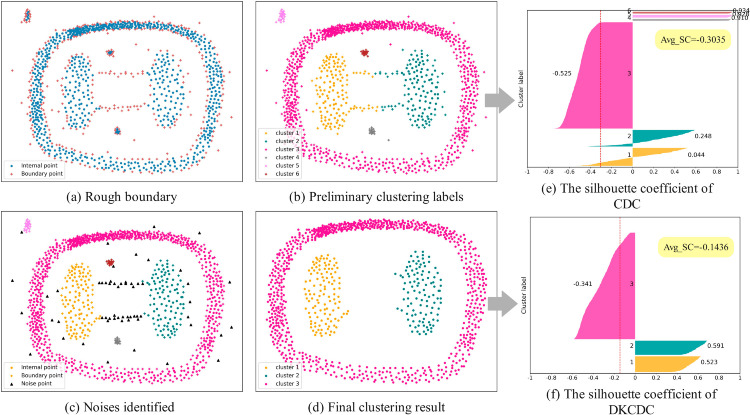
The four core steps of the DKCDC algorithm and the silhouette coefficients at different steps.

Fig 3 shows the four core steps of the DKCDC algorithm and the corresponding silhouette coefficients at different steps. Fig 3(a) depicts the rough boundaries generated based on the core idea behind CDC, which distinguishes boundary points and internal points according to the distribution of KNN. Obviously, in the datasets with bridge points and deviating points, the boundary search method based on KNN distribution can easily mark all the points on the bridge, the points that obviously deviate from the cluster, and points that are less obviously deviated at the edge of clusters as boundary points. Fig 3(b) presents the preliminary cluster labels obtained by clustering the boundary points and internal points through CDC. Obviously, since CDC ignores the judgment of the rationality of the boundary, points located on bridge structures and the deviation points are misclassified into neighboring clusters, which deteriorates the clustering accuracy. As illustrated in Fig 3(e), due to the unreasonable assignment of points located on bridge structures and the deviation points, the majority of these points are incorrectly classified into cluster 1 and cluster 2. This misclassification leads to the presence of sample points with negative silhouette coefficients within cluster 1 and cluster 2, thereby reducing the overall clustering performance. In addition, the special structure of the ring-shaped cluster is the main reason that the silhouette coefficients of all sample points in cluster 3 are negative. The defect that CDC cannot handle small-scale clusters also causes the average silhouette coefficients of cluster 4, 5, and 6 to be high. To solve the defects of CDC and most algorithms, this paper proposes a DKCDC algorithm using a fusion strategy, which can accurately detect the noise contained in the boundary to obtain clustering results with more accurate boundaries. Fig 3(c) indicates the noises identified using the fusion strategy. As shown in the figure, the black triangles denote noise points. It can be observed that under the fusion strategy, points on the bridge, points that are obviously deviated from the cluster and points that are less obviously deviated at the edge of clusters, which are contained within the boundary, are identified as noise, thereby making the cluster boundaries more clarity. Fig 3(d) presents the final clustering result after removing noise points. Combined with Fig 3(f), it is evident that since DKCDC identifies the points on the bridge structure and the deviation points as noise points and eliminates them, the interference caused by the noise points is avoided. All sample points are divided into the correct clusters, so that there are no sample points with negative silhouette coefficients in cluster 1 and cluster 2, and the silhouette coefficient of the ring-shaped cluster (cluster 3) is significantly improved. Moreover, DKCDC effectively identifies small-scale clusters (clusters 4, 5, and 6) as noise and eliminates them, thereby improving the overall clustering performance, and the silhouette coefficient is improved by 35.05% compared with CDC.

### Fusion strategy

The core of the DKCDC algorithm proposed in this paper is the adoption of a fusion strategy that combines the voting method and the distance method. Voting method is a commonly used decision-making approach in ensemble learning. The basic idea is to determine the final classification result based on the principle of minority obeying majority. In this paper, the category of a given point is preliminarily determined by employing a voting method to counts the number of samples from different categories within its r-domain. This method can effectively introduce local structural information, thereby enhancing the stability and robustness of clustering boundaries, especially in scenarios with imbalanced category distributions or the presence of noise points, and can avoid the interference caused by noise. In machine learning, distance measurement is an important tool for measuring the similarities or differences between samples. Common distance measurement methods include Euclidean distance, Manhattan distance, and Chebyshev distance. Distance method adopted in this paper integrates the Euclidean distance measurement to classify the boundary points by judging the difference between the distance from a boundary point to the nearest internal point among its K-nearest-neighbors and the average distance between the internal points in its r-domain. Based on the voting method, this method can further effectively identify the inconspicuous noise on the edge of the cluster.

The distinct of the fusion strategy is that it makes up for the fact that most algorithms, including those focused on boundary detection, ignore the rationality of the boundary. It combines the voting method with the distance method to obtain clearer clustering boundaries by detecting potential noise within the boundary. To achieve boundary refinement, the number of internal points and boundary points in the r-domain of the boundary point is combined with the distance from the point to its nearest internal point to divide it into more obvious deviation points, less obvious deviation points on the edge of the cluster, and points on the bridging structure.

The fusion strategy operates by evaluating each boundary point based on the number of internal point within its r-domain and distance to the K-nearest internal points. Points exceeding the majority voting threshold and exhibiting large neighbor distances are classified as noise. Given a dataset *G*(*P*), the CDC algorithm is used to obtain the preliminary clustering labels *Clust*(*P*), as well as the boundary point set *Bound*(*P*) and the internal point set *Inter*(*P*). For any boundary point $p_{i}\in Bound(P)$, a voting method is applied within its r-domain to count the number of internal points, which is denoted as $\left||Inter\_R(p_{i})|\right|$, and the number of boundary points, which is denoted as $\left||Bound\_R(p_{i})|\right|$, the internal point set within its r-domain is denoted as $Inter\_R(p_{i})$, the boundary point set within its r-domain is denoted as $Bound\_R(p_{i})$. At this time, the number of boundary points and internal points in the r-domain can be divided into three cases for discussion.

**Fig 4:**
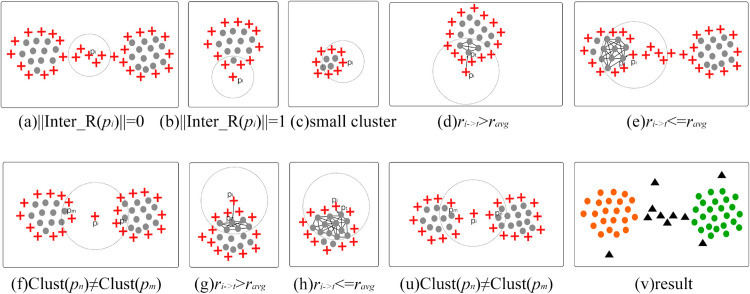
A fusion strategy that combines voting and distance methods. (a) The r-domain of the boundary point *p*_*i*_, which located on the bridge, does not include any internal points. (b) Boundary point *p*_*i*_ that deviates from cluster has its r-domain containing one internal point. (c) In small-scale cluster, the r-domain of the boundary point *p*_*i*_ contains most of the boundary points. (d) The r-domain of the boundary point *p*_*i*_, which deviates from the cluster, contains more boundary points, and $r_{i\to t}>r_{avg}$. (e) The number of boundary points within the r-domain of the boundary point *p*_*i*_, which located on the cluster’s edge, is greater than the number of internal points, and $r_{i\to t}\leq r_{avg}$. (f) The number of boundary points within the r-domain of the boundary point *p*_*i*_, which deviates from the cluster, is greater than the number of internal points and there are cross-cluster internal points. (g) The r-domain of the boundary point *p*_*i*_, which deviates from the cluster, contains more internal points, but $r_{i\to t}>r_{avg}$. (h) The number of boundary points within the r-domain of the boundary point *p*_*i*_, which located on the cluster’s edge, is smaller than the number of internal points, but $r_{i\to t}\leq r_{avg}$. (u) The number of boundary points within the r-domain of the boundary point *p*_*i*_, which deviates from the cluster, is smaller than the number of internal points and there are cross-cluster internal points. (v) The result of clustering under fusion strategy.

When $||Inter\_R(p_{i})||=1$ or $||Inter\_R(p_{i})||=0$, this suggests that *p*_*i*_ is very likely to either deviate obviously from the cluster or be located at the center of a bridge (as shown in Fig 4(a)–4(b), therefore, *p*_*i*_ should be classified as a noise point, and included in the noise set, $Noise(P)\cup =p_{i}$.

When $\left||Bound\_R(p_{i})||\geq ||Inter\_R(p_{i})|\right|$, it indicates that there are only a few internal points surrounding the boundary point *p*_*i*_. Under such scenario, identifying *p*_*i*_ as a noise point may result in the misclassification of some true boundary points as noise. (Take Fig 4(c) as an example, for small clusters with very few sample points, although the boundary points are densely distributed at the edge of the cluster, the number of internal points is significantly smaller than the number of boundary points, resulting in the number of boundary points in the r-domain of the boundary points exceeds that of internal points. In such a scenario, it is inappropriate to identify the boundary points as noise.) Therefore, to avoid some true boundary points are identified as noise, the algorithm proposed in this paper has taken measures that incorporate the distance method. For any boundary point *p*_*i*_, find the nearest internal point $p_{t}\in Inter\_R(p_{i})$ in its r-domain, and denote the distance from *p*_*i*_ to *p*_*t*_ as $r_{i\to t}$. The average distance between internal points in the r-domain of *p*_*i*_ is denoted as $r_{avg}$. As shown in Fig 4(d), if $r_{i\to t}>r_{avg}$, it indicates that the boundary point *p*_*i*_ deviates from the cluster and should be classified as a noise point, $Noise(P)\cup =p_{i}$; Otherwise it is false noise, as shown in Fig 4(e), which should be included in the internal points. It is essential to recognize that an overly large value for *r* could result in the presence of cross-cluster internal points within the r-domain of the boundary points, which will make an inflated calculation of $r_{avg}$, compromising the accuracy of judgments made about these boundary points. Therefore, before applying the distance method, it is necessary to combine the preliminary clustering labels obtained by the CDC algorithm to determine whether the Eq (1) holds. Taking Fig 4(f) as an example, there are cross-cluster internal points in the r-domain of the boundary point *p*_*i*_ between two clusters, so *p*_*i*_ should be classified as noise.

$$\forall p_{n},p_{m}\in Inter\_R\left(p_{i}\right),Clust\left(p_{n}\right)=Clust\left(p_{m}\right)$$ (1)

When $\left||Bound\_R(p_{i})||<||Inter\_R(p_{i})|\right|$, it indicates that there are only a few boundary points surrounding the boundary point *p*_*i*_. Directly classifying *p*_*i*_ as a true boundary point, it may result in the neglect of unobvious deviation points that are close to the cluster. Therefore, in such scenarios, it is essential to employ the distance method for further determination. If Eq (1) is established, it indicates that the internal points of *p*_*i*_ belong to the same cluster. In this case, *p*_*i*_ is a potential boundary and requires further determination using the distance method. Taken Fig 4(g)–4(h) as example, if the distance from *p*_*i*_ to its nearest internal point $p_{t}\in Inter\_R(p_{i})$, denoted as $r_{i\to t}$, exceeds the average distance $r_{avg}$ between internal points in the r-domain of *p*_*i*_, it indicates that *p*_*i*_ does not deviate significantly from the cluster and should be regarded as a false boundary, that is noise point. Otherwise, *p*_*i*_ is identified as a true boundary. If Eq (1) is not established (as shown in Fig 4(u)), then *p*_*i*_ is a noise point.

This fusion strategy, which combines the voting method and distance method, accurately detects noise within the boundary points, leading to more genuine and reliable boundary definitions and significantly improving clustering quality.

### Adaptive parameter adjustment

**Fig 5:**
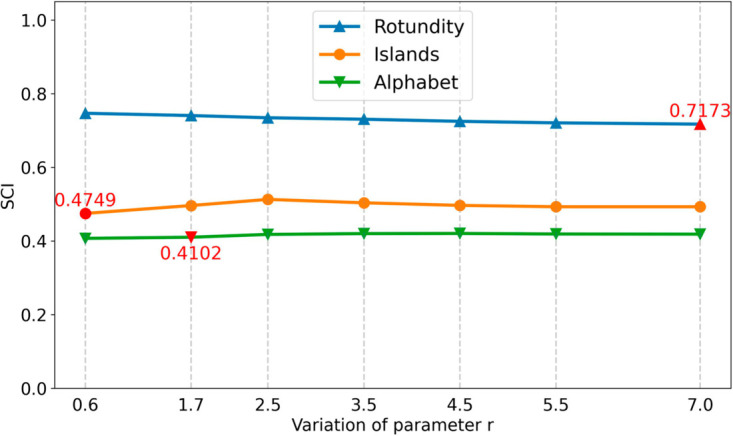
The silhouette coefficient obtained by the DKCDC algorithm under different parameters *r*.

**Fig 6:**
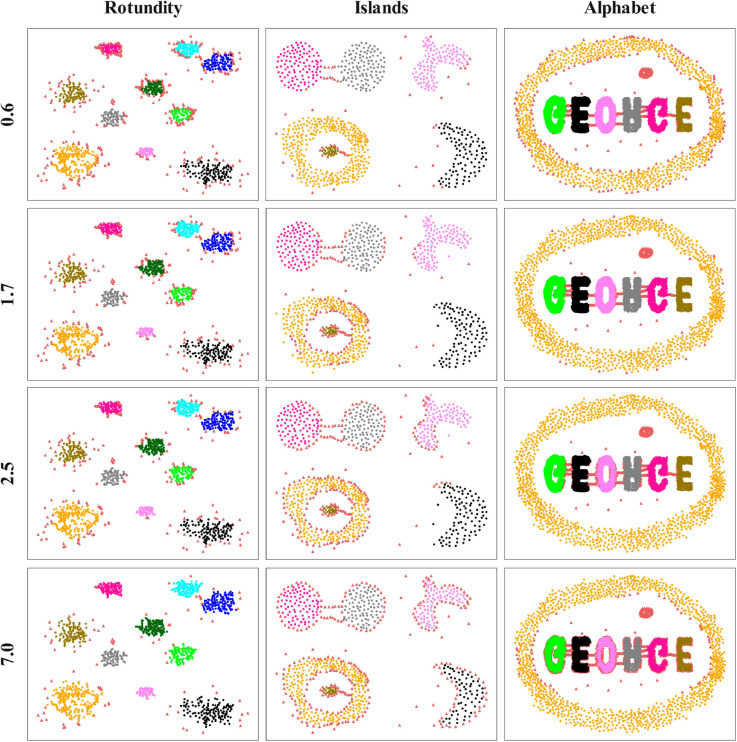
The noise distribution under different *r* values.

In this section, multiple experiments are conducted on three synthetic datasets and SCI is adopted as the evaluation index (as shown in Fig 5). In addition, the noise distribution, which is obtained by the fusion strategy under different *r* values, is visualized (as shown in Fig 6) to evaluate the impact of *r* values on the clustering effect. In a dataset, since noise points are typically located at the edge of clusters or between clusters, this ambiguous position makes them distant from other points in the cluster, or not obviously far away from other clusters, resulting in low intra-cluster similarity or high inter-cluster ambiguity. Consequently, the silhouette coefficient of the noise points is often low or even negative, after removing the noise points in dataset, the overall average silhouette coefficient will be improved.

Different *r* values will significantly affect the noise detection results in the fusion strategy. Improper selection of *r* can lead to misclassification of true boundary points as noise, thereby the number of detected noise points being greater than the actual value. Although eliminating more noise points has improved the overall silhouette coefficient to a certain extent, the actual clustering effect decreases. Using the Islands dataset in Fig 6 as an example, although increasing the parameter *r* slightly improves the silhouette coefficient, only the setting *r* = 0.6 enables accurate identification of both bridge structure and deviation points as noise. When *r* = 1.7 or 2.5, the true boundary points at the edges of both the two spherical clusters and the ring-shaped clusters are erroneously detected as noise. This incorrect detection of noise points will result in valid regions being excluded from region recognition processes, which may affect practical applications.

Therefore, this paper proposes an adaptive parameter adjustment function based on the K-nearest-neighbor distances between internal points in the dataset, which can automatically calculate the optimal *r* value, improve the parameter adjustment efficiency, and reduce the subjectivity caused by human intervention. First, the CDC algorithm is applied to obtain the result of preliminary clustering, N_Clust is denoted as the number of clusters, and the i-th cluster denoted as $C_{i}(i=1,2,...N\_Clust;)$. *Inter*(*C*_*i*_) represents the set of all internal points within the i-th cluster. For any *C*_*i*_, calculate the average distance of each internal point $p_{t}\in Inter(C_{i})$ to its k-nearest internal points, which belong to the same cluster, the average distance denoted as $Avg_{m}(p_{t})$, its definition is shown in Eq (2). Finally, as shown in Eq (3), the mean distance of all points in *C*_*i*_, which denoted as $\overset{―}{C_{i}}$, is calculated based on $Avg_{m}(p_{t})$, where n is the total number of internal points in *C*_*i*_. For these clusters, to obtain the final value of *r*, the maximum value of $\overset{―}{C_{i}}$, denoted as *max*(*r*), is computed, and then adjusted using a weighting coefficient.

**Algorithm 1. Adaptive parameter adjustment function.**

**Require:** *C*_*i*_, Inter(P), Dist_all(P), Knn(P), N_Clust

**Ensure:** The value of r

1: max(r)←0

2: **for** i∈N_Clust **do**

3:   n←0

4:   $∥Avg_{m}(p_{t})∥\leftarrow 0$

5:   $\overset{―}{C_{i}}\leftarrow 0$

6:   **if** Point $p_{t}\in Inter(C_{i})$ **then**

7:    n←n+1

8:    $∥dist(p_{t})∥\leftarrow 0$

9:    m←0

10:    $Avg_{m}(p_{t})\leftarrow 0$

11:    **if** Point $p_{b}\in Knn(p_{t})$ and $p_{b}\in Inter(C_{i})$ **then**

12:     m←m+1

13:     $∥dist(p_{t})∥\leftarrow ∥dist(p_{t})∥+Dist\_all[p_{t}][p_{b}]$

14:    **end if**

15:    $Avg_{m}(p_{t})\leftarrow \frac{1}{m}∥dist(p_{t})∥$

16:    $∥Avg_{m}(p_{t})∥\leftarrow ∥Avg_{m}(p_{t})∥+Avg_{m}(p_{t})$

17:   **end if**

18:   $\overset{―}{C_{i}}\leftarrow \frac{1}{n}∥Avg_{m}(p_{t})∥$

19:   **if** $\overset{―}{C_{i}}>max(r)$ **then**

20:     $max(r)\leftarrow \overset{―}{C_{i}}$

21:   **end if**

22: **end for**

23: **if** max(r)<2 **then**

24:   r←max(r)+0.3

25: **else**

26:   r←max(r)+1

27: **end if**

28: **return** r

$$Avg_{m}\left(p_{t}\right)=\frac{1}{m}\sum_{b=1}^{m}dist\left(p_{b},p_{t}\right)\;p_{b}\in Knn\left(p_{t}\right)\cap Inter\left(C_{i}\right)$$ (2)

$$\overset{―}{C_{i}}=\frac{1}{n}\sum_{t=1}^{n}Avg_{m}\left(p_{t}\right)$$ (3)

According to $\overset{―}{C_{i}}$, the density of the distribution of sample points in cluster i can be judged. If $\overset{―}{C_{i}}<2$, it indicates that cluster i is a relatively dense cluster, at this time, the weighting coefficient of parameter *r* is set to 0.3. This is because, in dense clusters *C*_*i*_, the weighting coefficient of *r* lower than 0.3, suggesting that *r* is too small, may result in neglecting noise hidden within boundary points. On the contrary, if the weighting coefficient of *r* is greater than 0.3, suggesting that *r* is too large, it will cause some true boundaries to be incorrectly identified as noise. If $\overset{―}{C_{i}}\geq 2$, it indicates that cluster i is a relatively sparse cluster, at this time, the weighting coefficient of parameter *r* is set to 1.

### DKCDC algorithm flow

**Fig 7:**
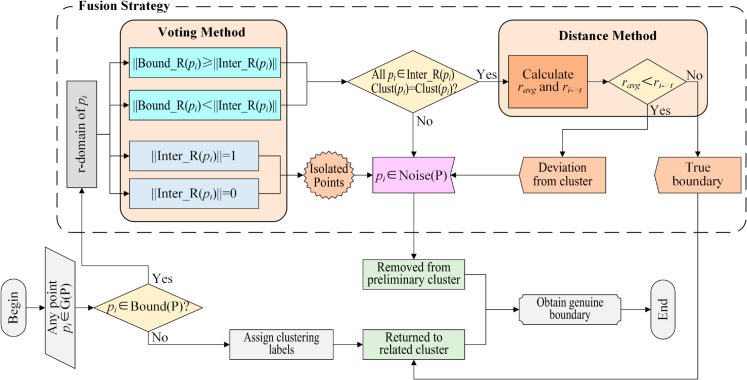
The overall flow chart of DKCDC algorithm.

Fig 7 illustrates the overall flow chart of the DKCDC algorithm. Given a dataset *G*(*P*), the original CDC algorithm is employed to distinguish the boundary points and internal points, and to generate preliminary clustering labels. For each point in the dataset, determine whether it belongs to the boundary. If it is not a boundary point, keep its preliminary assigned label. If the point is identified as a boundary point, it may be potential noise and needs to be further judged using a fusion strategy. If it is not a noise, then false noise *p*_*i*_ is returned to the corresponding cluster as a true boundary. If it is a noise point, then true noise *p*_*i*_ is removed from its preliminary assigned cluster, to achieving a genuine boundary.

The specific process of the fusion strategy is also shown in Fig 7. In the r-domain of boundary point *p*_*i*_, a voting method is employed. When there are no internal points or only one internal point within r-domain, as shown in Fig 4(a)–4(b), this indicates that *p*_*i*_ is isolated point and should be classified as noise. When the number of internal points in the r-domain of *p*_*i*_ lower than that of boundary points, the first step is to examine if there are cross-cluster internal points within this domain, the existence of such points suggests that *p*_*i*_ is likely located between two clusters and should be added to the noise point set, as shown in Fig 4(f). In the absence of cross-cluster internal points, the distance method is used to determine the positional relationship between *p*_*i*_ and the cluster. When $r_{i\to t}\leq r_{avg}$, this suggests that *p*_*i*_ is located on the cluster edge, thus classifying it as a true boundary point, as shown in Fig 4(e). When $r_{i\to t}>r_{avg}$, this suggests that *p*_*i*_ is the point that is obviously deviated from the cluster, thus classifying it as a noise, as shown in Fig 4(d). Similarly, when the number of internal points in the r-domain of *p*_*i*_ is greater than the number of boundary points, if there are cross-cluster internal points in the domain, *p*_*i*_ is classified as a noise point, as shown in Fig 4(u). If there are no cross-cluster internal points, further evaluation is performed using the distance method. When $r_{i\to t}\leq r_{avg}$, it is indicated that *p*_*i*_ is distributed at the edge of the cluster and belongs to the true boundary, as shown in Fig 4(h). When $r_{i\to t}>r_{avg}$, it is indicated that *p*_*i*_ is the point that is less obviously deviated at the edge of clusters, it is necessary to identify *p*_*i*_ as a noise point, as shown in Fig 4(g).

The fusion strategy, which combines the voting method and distance method, effectively identifies noise points concealed within boundary points, leading to an effective improvement in clustering accuracy of DKCDC algorithm.

### Time complexity analysis of DKCDC

The time complexity of the DKCDC algorithm proposed in this paper consists of the following three parts:

1. The time complexity of computing the distance matrix, denoted as $Dist\_all_{n*n}$, for all pairs of points in the dataset is *O*(*n*^2^).
2. The complexity of adaptive parameter adjustment for parameter *r* is *O*(*n*).
3. The complexity of the fusion strategy is *O*(*n*^3^).

In conclusion, the overall time complexity for the DKCDC algorithm is *O*(*n*^3^), whereas the time complexity for the traditional CDC algorithm is *O*(*n*^2^). Mathematically, the time complexity of the CDC algorithm is more favorable than that of the DKCDC algorithm; however, when applied to complex datasets that include bridges and outlier points, the clustering efficacy of the DKCDC algorithm exceeds that of the CDC algorithm.

## Results and discussion

To comprehensively evaluate the clustering performance of the proposed DKCDC algorithm, this section selects three representative clustering algorithms as baselines and conducts experimental comparisons on different datasets. Firstly, experiments are conducted on three artificial datasets with typical structural characteristics, and the clustering performance is evaluated by the Silhouette Coefficient Index (SCI). The results demonstrate that the proposed DKCDC algorithm achieves superior clustering accuracy. Subsequently, on three UCI benchmark datasets with true labels, the clustering accuracy (ACC), Fowlkes-Mallows Index (FMI), and Adjusted Rand Index (ARI) are used as evaluation indicators to further demonstrate that the clustering results generated by the DKCDC algorithm have higher reliability and stability in label consistency.

### Cluster evaluation index

SCI is one of the important index to measure the rationality of clustering. It is mainly used to evaluate the cohesion of samples within clusters and the separation from other clusters. It is defined as follows:

$${SC}_{i}=\frac{y_{i}-x_{i}}{\max(x_{i},y_{i})}$$ (4)

Where *x*_*i*_ represents the cohesion within cluster, *y*_*i*_ represents the separation of inter cluster. For each sample, the SCI value range is [–1,1]. A value closer to 1 indicates a more appropriate sample division, while a negative value suggests that the sample may have been misclassified. In cluster analysis, a higher average SCI value usually means a clearer clustering result.

ACC is an index that measures the matching degree between clustering results and real labels. The core idea is to calculate the correct prediction ratio of samples by measuring the degree of match between cluster labels and real labels. The ACC value range is [0,1], a higher value indicates a better agreement between the clustering result and the real labels.

FMI measures the similarity between two clustering results. It is computed as the geometric mean of precision and recall derived from the clustering result and real labels. The value ranges is [0,1], with higher values indicating better clustering performance.

ARI is used to evaluate the similarity between clustering results and real classification. The core idea is to compare the clustering consistency of sample pairs in different clustering results. The value of ARI is from –1 to 1, where negative values indicate a significant mismatch between the clustering results and the real labels, 0 represents a random result, and 1 represents a complete match.

### Experimental results on artificial datasets

**Fig 8:**
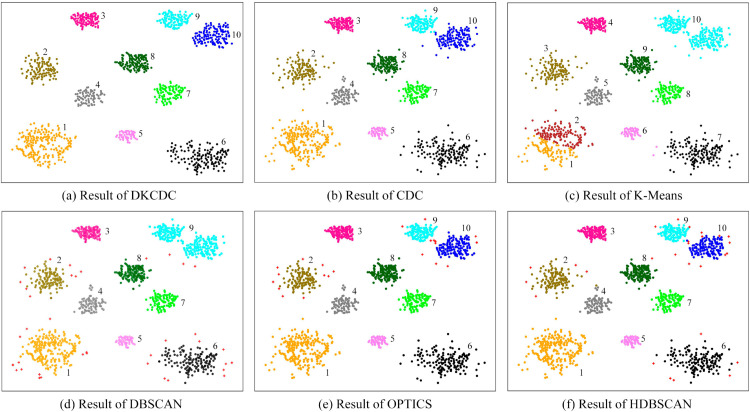
The results of DKCDC, CDC, K-Means, DBSCAN, OPTICS, HDBSCAN algorithms on Rotundity dataset: (a)-(f).

**Fig 9:**
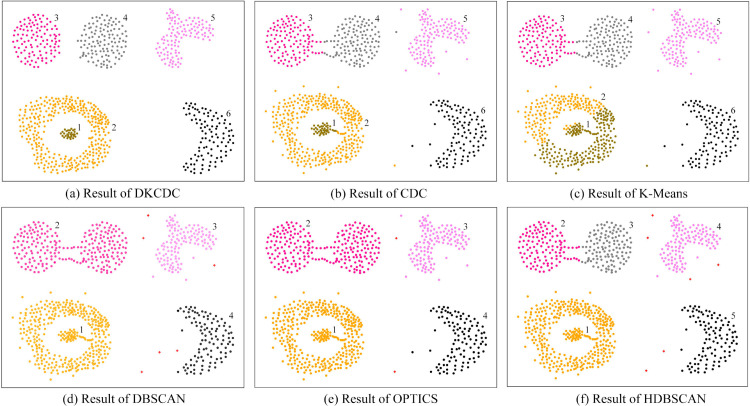
The results of DKCDC, CDC, K-Means, DBSCAN, OPTICS, HDBSCAN algorithms on Islands dataset: (a)–(f).

**Fig 10:**
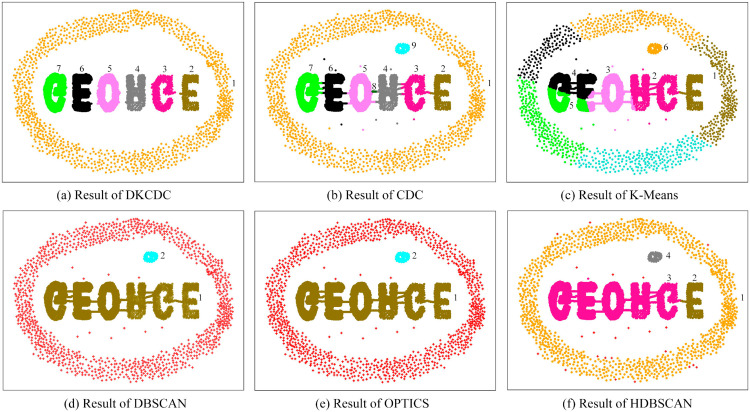
The results of DKCDC, CDC, K-Means, DBSCAN, OPTICS, HDBSCAN algorithms on Alphabet dataset: (a)–(f).

To evaluate the universality and robustness of the proposed DKCDC algorithm, comparative experiments were conducted on three artificial datasets with varying data structures. The silhouette coefficient was utilized as a measurement index to compare the clustering performance of four different algorithms across these three distinct datasets. The performance evaluation results of the silhouette coefficient are shown in Table 1 (The bold data in the table represent the best evaluation results), the clustering results on three different datasets are shown in Figs 8, 9, and 10.

**Table 1:** Comparison of clustering results on artificial datasets (Use the Silhouette Coefficient as measurement index).

Dataset	DKCDC(*ours*)	CDC	K-Means	DBSCAN	OPTICS	HDBSCAN
Rotundity	**0.7173**	0.6826	0.6245	0.6491	0.6658	0.6596
Islands	0.4749	0.2781	0.4955	0.5658	**0.5822**	0.5676
Alphabet	**0.4102**	0.1596	0.3911	0.3193	0.3193	0.3240

In Table 1, the DKCDC algorithm proposed in this paper demonstrates an improvement of at least 5.08% in the silhouette coefficient compared to the other five algorithms on the Rotundity dataset. And on the Alphabet dataset, the silhouette coefficient of DKCDC increased by at least 4.88%. On the Islands dataset, despite DKCDC fails to achieve the optimal silhouette coefficient, it is significantly better than that of the original CDC. Additionally, as shown in Fig 9, the clustering performance of K-Means, DBSCAN, OPTICS and HDBSCAN on this dataset is inferior to that of DKCDC.

In Fig 8, the Rotundity dataset consists of 10 spherical clusters with varying densities, including both points markedly deviating from cluster centers and marginally deviating cluster edge points. The Rotundity dataset, which includes spherical clusters with varying densities and diverse deviating points, makes a challenging for clustering on this dataset. Therefore, this dataset provides an opportunity for a comprehensive assessment of the DKCDC algorithm, which enhances boundary precision by detecting noise points, better demonstrates its performance in handling of noise points that deviate from the cluster. As observed in Fig 8, all algorithms except DBSCAN partitioned the dataset into 10 clusters. However, as shown in Fig 8(c), the K-Means algorithm mistakenly divides the cluster in the bottom left into two separate parts. Since K-Means is unable to block the connection between boundary points of different clusters by identifying boundaries, the two clusters that are close to each other in the upper right corner are mistakenly classified as a single category. Obviously, the clustering effect of K-Means on this dataset is unsatisfactory. In Fig 8(b), although the CDC algorithm can block the connections between boundary points of different clusters by identifying boundaries, it fails to accurately detect noise points. As a result, the deviating points contained within the boundary points are not handled, making it difficult to obtain precise boundaries. Therefore, those deviating points scattered around different clusters are mistakenly classified as in-cluster points, which reduces the silhouette coefficient. The DKCDC algorithm proposed in this paper addresses the defect of the CDC algorithm and most other methods, which struggle to obtain a more precise boundary. The DKCDC algorithm can detect points that deviate from the cluster based on the average distance between internal points, subsequently identifying them as noise for removal. Therefore, those deviation points can be accurately identified as noise points, enhancing the precision of the boundary. As shown in Fig 8(a), under the DKCDC algorithm, the separation between clusters is clearer and exhibits greater accuracy, resulting in a significant improvement in the silhouette coefficient, which reaches 0.7173, compared to the other five algorithms. Fig 8(d) is the result obtained under DBSCAN, where the red ‘+’ denotes noise. Obviously, the algorithm can only identify points that are obviously deviated from the cluster as noise, while the points that are less obviously deviated at the edge of clusters are difficult to detect, resulting in the two clusters that are close to each other in the upper right corner being mistakenly identified as one class. Therefore, DBSCAN fails to produce satisfactory clustering results. Fig 8(e) shows the result obtained by OPTICS, this algorithm overcomes the weak connectivity disadvantage of DBSCAN and successfully identifies two clusters that are close to each other in the upper right corner, but ignores some points that are obviously deviated from the cluster center. In Fig 8(f), since HDBSCAN constructs the clustering structure based on density-reachable graphs, those points that deviate from the cluster in space but still have density reachability cannot be detected as noise.

In Fig 9, the Islands dataset contains two spherical clusters connected by multiple bridges, an arbitrarily shaped cluster, a crescent-shaped cluster, and an isolated cluster surrounded by a ring with a bridge. There are also some points that obviously deviated from the clusters are dispersed around different clusters. The diversity of clusters in the Islands dataset provides a meaningful study for clustering, not only showing how different clustering algorithms handle ring-shaped clusters, but also how they handle clusters with bridges and outliers. In Fig 9(b), the CDC algorithm divides the dataset into 6 categories. Since the CDC algorithm cannot accurately detect noise within the boundary points to achieve precise boundaries, the points on the bridge and the points obviously deviate from clusters, which are included within the boundary points, are mistakenly classified as in-cluster points, making it impossible to block the connection of boundary points between clusters connected by bridges. As shown in Fig 9(a), the DKCDC algorithm proposed in this paper takes into account the rationality and precision of the boundary. This algorithm identifies the points on bridge and the points obviously deviate from clusters, which are contained within the boundary, as noise and removes them. This process results in more accurate boundaries, effectively blocking single and multiple bridge connections, and avoiding the interference from scattered deviating points, thereby significantly improving the silhouette coefficient. In Fig 9(c), the K-Means algorithm performs poorly in clustering the isolated cluster surrounded by the ring and is unable to identify noise points. Since K-Means is not a clustering algorithm based on boundary search, it is impossible to block the connection of boundary points between clusters connected by bridges. The silhouette coefficient obtained by the DBSCAN algorithm can reach 0.5658, however, as shown in Fig 9(d), DBSCAN cannot accurately identify the boundary, resulting in the inability to separate clusters with weak connectivity, so that the two ring-shaped clusters connected by bridges and the island surrounded by a ring are classified into one category respectively. Although OPTICS and HDBSCAN achieve higher silhouette coefficients than DKCDC, as shown in Fig 9(e) and 9(f), both algorithms fail to handle bridging structures and points that deviate significantly from cluster centers. Even though HDBSCAN identifies the two spherical clusters connected by bridges, it assigns the bridge points directly to the adjacent clusters, which affects the clustering quality. Therefore, although the silhouette coefficient of DKCDC on the Islands dataset is slightly lower than that of some algorithms, its clustering effect is significantly better than the other five algorithms, showing higher actual clustering quality.

In Fig 10, the Alphabet dataset includes six letter clusters connected by multiple bridges, a large ring, and a small spherical cluster. There are some points that are scattered inside the ring, which are obviously deviated from the cluster. And some inconspicuous deviation points that are close to the edge of the cluster are distributed around the ring. The complexity of the data distribution of this dataset provides an important opportunity to further study the performance of clustering algorithms. In Fig 10(b), the dataset is divided into 9 categories, however, cluster 9 only contains a relatively small number of points, and a small section of the bridge connecting the letters ‘O’ and ‘R’ is also classified as a separate category (cluster 8). Since the CDC algorithm cannot obtain a well-defined clustering boundary by accurately identifying noise, the points on bridge and the points with deviations from cluster centers, which are included in the boundary points, cannot be identified as noise, thus affecting the clustering results. Fig 10(a) is the result obtained by the DKCDC algorithm. Under the DKCDC algorithm, the dataset is divided into 7 clusters with relatively precise boundaries, and the points on bridge and the points with deviations from cluster centers, which are included in the boundary points, are removed as noise points. The small clusters were discarded, and the silhouette coefficient reached 0.4102, which was significantly better than the other five algorithms. As shown in Fig 10(c), the algorithm characteristics of K-Means make it only suitable for processing spherical clusters, consequently, it fails to recognize both ring-shaped clusters and irregularly shaped letter clusters. A comparison of Figs 10(d) and 10(e) reveals that DBSCAN and OPTICS produce similar clustering results on this dataset, this is because both algorithms rely on point density to form cluster structures. Although OPTICS improves upon DBSCAN by handling its poor performance on datasets with significant density variations, the Alphabet dataset lacks clusters with notable density differences, consequently, the algorithmic advantages of OPTICS cannot be reflected, and the two algorithms essentially degenerate into similar processing, resulting in output results that are extremely close to DBSCAN. Fig 10(f) shows the results obtained by HDBSCAN, which focuses on density reachability and cluster stability. The ring-shaped cluster is identifiable because all points lie in regions of similar density, and the reachability distances change smoothly. The letter ‘E’ on the right exhibits a significantly higher local density, so it forms a stable high-density cluster, while the other letters are sparsely distributed and have larger intervals, which makes them unstable and unable to merge into the right-side ‘E’. On the Alphabet dataset, DKCDC performed best in both clustering performance and evaluation indicators, achieving the highest silhouette coefficient and significantly outperforming other algorithms in clustering result quality.

The above analysis indicates that the DKCDC algorithm has higher clustering accuracy than the CDC algorithm, and the overall performance of the DKCDC algorithm on artificial datasets is better than the other five algorithms.

### Experimental results on UCI datasets

To further test the clustering performance of the DKCDC algorithm, the DKCDC algorithm and the other five algorithms were applied to several UCI datasets with different data structures and dimensions. The performance of different algorithms on the dataset is evaluated using three commonly employed external metrics.

**Fig 11:**
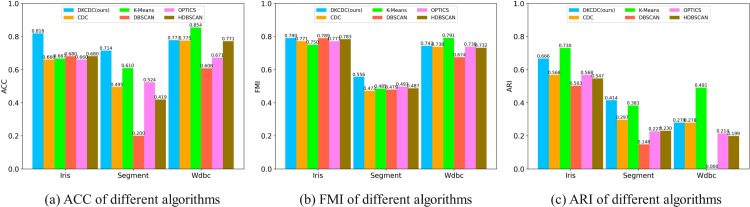
Cluster evaluation index comparison chart of different algorithms on UCI datasets: (a)–(c).

The data structure of the UCI dataset used in this experiment is shown in Table 2. The Iris dataset, which includes 150 samples and 4 numerical features, has 3 different categories. The number of samples in each category is equal, and there is some overlap between categories. The simplified Segment dataset contains 210 sample, 19 numerical features, and 7 different categories. The samples in each category are balanced, but some features may overlap between categories. The Wdbc dataset is one of the classic datasets used for breast cancer diagnosis. It contains 569 samples, 30 numerical features, and 2 different categories, and the category distribution is uneven. Overall, applying the DKCDC algorithm to these three datasets with differing characteristics is valuable for evaluating its effectiveness in high-dimensional spaces. In addition, three different indicators, ACC, FMI and ARI are used in this experiment to evaluate clustering performance. By employing these three evaluation indicators with distinct characteristics, a more comprehensive and scientific assessment of the DKCDC algorithm’s performance on high-dimensional datasets such as Iris, Segment, and Wdbc can be achieved. The clustering evaluation results are shown in Table 3 (the bold data in the table represent the best evaluation results) and Fig 11.

**Table 2:** UCI datasets.

Dataset	Sample	Dimension	Clusters
Iris	150	4	3
Segment	210	19	7
Wdbc	569	30	2

**Table 3:** Comparison of clustering results on UCI datasets.

Dataset	Index	DKCDC(*ours*)	CDC	K-Means	DBSCAN	OPTICS	HDBSCAN
Iris	ACC	**0.8182**	0.6600	0.6667	0.6800	0.6600	0.6800
	FMI	**0.7901**	0.7715	0.7505	0.7890	0.7715	0.7833
	ARI	0.6656	0.5681	**0.7302**	0.5026	0.5681	0.5468
Segment	ACC	**0.7143**	0.4952	0.6095	0.2000	0.5238	0.4190
	FMI	**0.5559**	0.4716	0.4851	0.4793	0.4972	0.4869
	ARI	**0.4145**	0.2966	0.3829	0.1477	0.2266	0.2298
Wdbc	ACC	0.7772	0.7750	**0.8541**	0.6081	0.6714	0.7715
	FMI	0.7432	0.7376	**0.7915**	0.6742	0.7378	0.7315
	ARI	0.2791	0.2787	**0.4914**	0.0000	0.2141	0.1985

In Table 3, in comparison to the other three algorithms, DKCDC exhibits an improvement of at least 17.19% in ACC on the Iris and Segment datasets, alongside a minimum increase of 0.14% in the FMI metric. Furthermore, on the Segment dataset, the ARI is enhanced by at least 8.25%. By analyzing the three external indicators of each algorithm across different datasets in Table 3, it can be observed that the DKCDC algorithm achieves at least a 0.14% improvement in clustering performance over the CDC. In the Iris dataset, the ACC obtained by DKCDC reaching 0.8182, which is significantly higher than other algorithms, this indicates that the clustering results under DKCDC match the real labels with an accuracy of 81.82%, that is, 81.82% of the data are assigned to the correct clustering labels. In addition, the FMI of DKCDC on Iris dataset is 0.7901, which demonstrates that the consistency between clustering results and real labels reaches 79.01%, slightly higher than other algorithms. However, under DKCDC, the similarity between the clustering results and the real classification can only reach 66.56%, that is, ARI=0.6656, which is slightly lower than the K-Means algorithm. This is due to the fact that the Iris dataset exhibits a relatively balanced distribution of categories, with the Setosa category being clearly distinguishable from the other two in the feature space. This linearly separable feature is advantageous for clustering using K-Means. While there is some dimensional overlap between the Versicolor and Virginica categories, the overall cluster shapes are relatively spherical. This characteristic suits the K-Means algorithm, which performs well with spherical clusters, thus resulting in a higher ARI. In the Wdbc dataset, the three evaluation indicators of the DKCDC algorithm are slightly higher than those of the CDC algorithm, but the K-Means algorithm achieves the best evaluation indicator on the Wdbc dataset. This is because the two categories in the Wdbc dataset exhibit significant differences across different features, showing a relatively spherical or symmetrical distribution in the feature space. The data structure is thus more suitable for effective clustering by distance-based algorithms like K-Means, resulting in superior performance on ACC, FMI, and ARI metrics.

Fig 11 clearly indicates that DKCDC achieves higher clustering performance than CDC on three high-dimensional UCI datasets, with improvements of at least 0.28%, 0.76%, and 0.14% in ACC, FMI, and ARI, respectively. This demonstrates the superior effectiveness of DKCDC in handling high-dimensional dataset.

## Conclusion

The clustering method that detects the boundary points of clusters to interrupt the connection between boundary points of different clusters can improve clustering accuracy. However, most clustering algorithms, including those based on boundary search, do not take into account the reasonableness and genuineness of boundaries, which is because that their primary aim not being regional division. Especially for datasets with bridges between clusters, although bridge points and points slightly deviations from cluster centers can be marked as boundary points, it remains challenging to accurately detect noise, making it difficult to achieve genuine boundary results. Therefore, this paper proposes a new boundary search clustering algorithm (DKCDC) that employs a fusion strategy, designed for regional division. First, a fusion strategy combining the number of votes and the K-nearest neighbor distance is adopted to identify the noise hidden within the boundary, thereby obtaining clustering results with clear boundaries, which makes up for the neglect of boundary genuine by CDC algorithm and most algorithms. Second, to avoid the subjective influence of artificial adjustment of the parameter *r* in the fusion strategy, an adaptive adjustment of the *r* value is set. Finally, the DKCDC algorithm proposed in this paper was compared with the CDC, K-Means, DBSCAN, OPTICS and HDBSCAN algorithms on artificial and UCI datasets. Results from the experiments reveal that the DKCDC has significant advantages in noise identification, and it achieves at least a 4.88% improvement in silhouette coefficient over other methods.

In future work, the following issues will be explored and studied. First, the application of the fusion strategy for computing distances among internal points presents a high level of complexity; therefore, a new distance calculation method will be developed to decrease the time complexity of the algorithm. Second, the DKCDC algorithm will be attempted to be extended to large-scale and high-dimensional datasets to improve the practical application capabilities of the clustering algorithm.
